# A model of human motor sequence learning explains facilitation and interference effects based on spike-timing dependent plasticity

**DOI:** 10.1371/journal.pcbi.1005632

**Published:** 2017-08-02

**Authors:** Quan Wang, Constantin A. Rothkopf, Jochen Triesch

**Affiliations:** 1 Frankfurt Institute for Advanced Studies, Ruth-Moufang Str. 1, 60438 Frankfurt, Germany; 2 Centre for Cognitive Science & Institute of Psychology, Technical University Darmstadt, Darmstadt, Germany; Ghent University, BELGIUM

## Abstract

The ability to learn sequential behaviors is a fundamental property of our brains. Yet a long stream of studies including recent experiments investigating motor sequence learning in adult human subjects have produced a number of puzzling and seemingly contradictory results. In particular, when subjects have to learn multiple action sequences, learning is sometimes impaired by proactive and retroactive interference effects. In other situations, however, learning is accelerated as reflected in facilitation and transfer effects. At present it is unclear what the underlying neural mechanism are that give rise to these diverse findings. Here we show that a recently developed recurrent neural network model readily reproduces this diverse set of findings. The self-organizing recurrent neural network (SORN) model is a network of recurrently connected threshold units that combines a simplified form of spike-timing dependent plasticity (STDP) with homeostatic plasticity mechanisms ensuring network stability, namely intrinsic plasticity (IP) and synaptic normalization (SN). When trained on sequence learning tasks modeled after recent experiments we find that it reproduces the full range of interference, facilitation, and transfer effects. We show how these effects are rooted in the network’s changing internal representation of the different sequences across learning and how they depend on an interaction of training schedule and task similarity. Furthermore, since learning in the model is based on fundamental neuronal plasticity mechanisms, the model reveals how these plasticity mechanisms are ultimately responsible for the network’s sequence learning abilities. In particular, we find that all three plasticity mechanisms are essential for the network to learn effective internal models of the different training sequences. This ability to form effective internal models is also the basis for the observed interference and facilitation effects. This suggests that STDP, IP, and SN may be the driving forces behind our ability to learn complex action sequences.

## Author summary

From dialing a phone number to driving home after work, much of human behavior is inherently sequential. But how do we learn such sequential behaviors and what neural plasticity mechanisms support this learning? Recent experiments on sequence learning in human adults have produced a range of confusing findings, especially when subjects have to learn multiple sequences at the same time. For example, the succes of training can strongly depend on subjects’ training schedules, i.e., whether they practice one task until they are proficient before switching to the next or whether they interleave training of the different tasks. Here we show that a model self-organizing neural network readily explains many findings on human sequence learning. The model is formulated as a recurrent network of simplified spiking neurons and incorporates multiple biologically plausible plasticity mechanisms of neurons and synapses. Therefore, it offers a theoretical bridge between basic mechanisms of synaptic and neuronal plasticity and the behavior of human subjects in sequence learning tasks.

## Introduction

Humans can improve their performance in sequential movement tasks through practice, but such motor learning has shown puzzling and seemingly contradictory results. On the one hand, a wide variety of proactive and retroactive interference effects have been observed when multiple tasks have to be learned [1]. On the other hand, some studies have reported facilitation and transfer of learning between different tasks, sometimes based on abstract structure similarities [2]. At present it is unclear what learning mechanisms give rise to these various findings, how these effects depend on the training, what their biophysical substrate is, and in what brain structures they are implemented.

Progress towards answering questions about the neural underpinnings of sequence learning in humans and other mammals has revealed that it depends on a number of brain structures including the recurrent loops between neocortex, cerebellum, and basal ganglia [3]. At this system level, computational modeling work rooted in reinforcement learning has tried to explain the contributions of different brain areas [4] while matching the behavioral performance of humans and monkeys. At the cellular level, there has been a strong interest in how the learning of sequential patterns may be supported by the temporally asymetric learning window of spike-timing-dependent plasticity (STDP) [5–9] and related learning rules, e.g., [10–17], review in [18]. Furthermore, it has been investigated how the relatively short time windows associated with STDP might be extended to behaviorally relevant time scales [19]. However, such models have not been related to human performance in actual sequence learning experiments and no mechanistic explanation of the above-mentioned interference and facilitation effects has been given.

Here we show how these effects can be understood based on the interaction of different learning mechanisms in a recurrent neural network model. Specifically, we consider the self-organizing recurrent neural network (SORN), a sparsely connected recurrent network model whose activity and connectivity are shaped by three plasticity mechanisms: spike timing-dependent plasticity (STDP), intrinsic plasticity of neuron excitability, and a form of synaptic normalization [20]. Despite its simplicity, the original SORN model and a recent extension have been shown to exhibit powerful sequence learning abilities [20, 21]. Moreover, a variation of the SORN has been shown to match findings on the probability distribution and the pattern of fluctuations of synaptic efficacies in neocortex and hippocampus [22]. Most recently, it has been shown that the SORN can reproduce a range of findings on neural spiking variability and the relationship between spontaneous and evoked activity patterns [23]. Therefore, it is an interesting candidate model for trying to bridge the gap between behavioral performance of human subjects on the one hand and cellular and synaptic mechanisms of plasticity on the other hand.

In the present work, we consider a SORN model which receives stimulus-specific input and is connected to a layer of motor neurons mediating movement sequences through a winner-take-all mechanism. We use this network to model a series of experiments on movement sequence learning [1, 24–26] using a single set of parameters in all simulations. We furthermore show the robustness of these results across variations of network parameters. The network learns to carry out the correct movement sequences over trials and reproduces differences in behavior between training schedules such as blocked vs. randomly interleaved training. The network also reproduces human performance in tasks with similar training sequences but different training times. In addition, it shows how psychophysical performance measures are reflective of the learned neuronal representations in the recurrent network. Mutual information calculations and PCA of network activity reveal how input representations and trajectories of neural activity change with training. Importantly, by parametrically varying tasks when learning multiple sequences we find an interaction between training schedule and task similarity, which provides testable predictions for further experiments. In sum, we show how fundamental mechanisms of neural plasticity may be responsible for the rich set of interference and facilitation effects induced by task similarity and training schedule in human sequence learning.

## Methods

### Self-organizing recurrent neural network model

**Fig 1:**
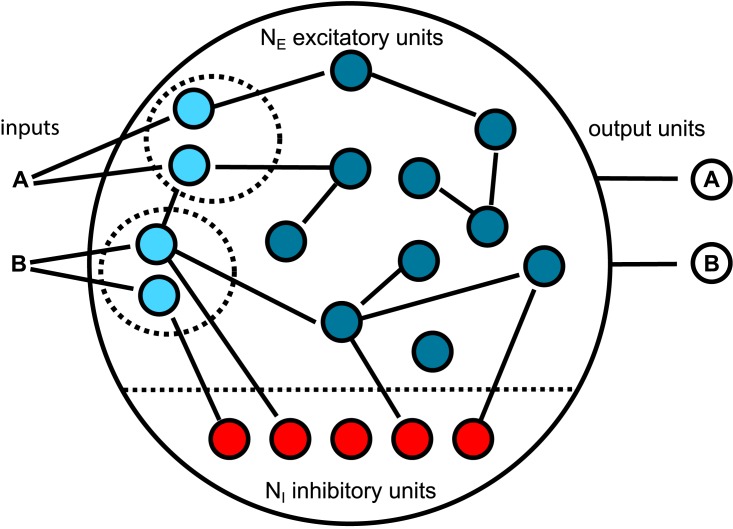
Structure of Self-Organizing Recurrent Neural Network (SORN). Input units (cyan) directly receive external input in a non-overlapping way and connect to other excitatory reservoir units (blue). Excitatory reservoir units are also connected to inhibitory units, as well as the output units. The weights between the reservoir units and the output units are trained with supervised methods.

In this section, we present a specific recurrent neural network with threshold units combining three different forms of plasticity. The network architecture here belongs to the SORN family: a self-organizing recurrent neural network [5, 20, 22, 23] and a schematic is provided in Fig 1. In contrast to traditional reservoir computing architectures [27–29] the “reservoir” is not static in SORNs but adapts to inputs via multiple plasticity mechanisms giving rise to powerful sequence learning abilities [20, 21].

The network is composed of *N*_*E*_ excitatory (*E*) and *N*_*I*_(= 0.2 × *N*_*E*_) inhibitory (*I*) threshold units. Neurons are connected through weighted synaptic connections, where *W*_*ij*_ is the connection strength from unit *j* to unit *i*, with self-connections being prohibited. All possible connections between the excitatory and inhibitory neuron populations are present (*W*^*IE*^ and *W*^*EI*^), while the excitatory to excitatory connections *W*^*EE*^ are sparse and random. On average each neuron has λ_*W*_ incoming and outgoing connections. Direct connections between inhibitory units are absent. The initial weight strengths are drawn from the interval [0, 1] and subsequently normalized such that the incoming connections to a neuron sum up to one: $\sum_{j}W_{ij}^{IE}=1$, $\sum_{j}W_{ij}^{EI}=1$, $\sum_{j}W_{ij}^{EE}=1$.

The network state, at a discrete time *t*, is given by the binary vectors *x*(*t*) with length *N*_*E*_ and *y*(*t*) with length *N*_*I*_ corresponding to the activity of the excitatory and inhibitory units, respectively. The *T*_*E*_ and *T*_*I*_ are threshold values for the excitatory and inhibitory units. They are initially drawn from a uniform distribution in the interval $\left[0,T_{max}^{E}\right]$ and $\left[0,T_{max}^{I}\right]$, respectively. The Heaviside step-function *θ*(.) constrains the activation of the network at time *t* to a binary representation: The neuron *i* fires if the total drive it receives is greater then its threshold (*x*_*i*_(*t*) = 1) otherwise it stays silent (*x*_*i*_(*t*) = 0). The evolution of the network state is described by:

$$\begin{array}{c} x_{i}\left(t+1\right)=\Theta (\sum_{j=1}^{N_{E}}W_{ij}^{EE}\left(t\right)x_{j}\left(t\right)-\sum_{k=1}^{N_{I}}W_{ik}^{EI}y_{k}\left(t\right)+v_{i}^{U}\left(t\right)-T_{i}^{E}\left(t\right)) \end{array}$$ (1)

$$\begin{array}{c} y_{i}\left(t+1\right)=\Theta (\sum_{j=i}^{N_{E}}W_{ij}^{IE}x_{j}\left(t+1\right)-T_{i}^{I}). \end{array}$$ (2)

Each input symbol (letter or digit) is associated with a predefined subset of *N*^*U*^ input units, and all neurons *i* in the corresponding group will receive a positive input drive ($v_{i}^{U}\left(t\right)=1$). There is no overlap between input units of different symbols.

We are using the same plasticity mechanisms as the original SORN. The network relies on three forms of plasticity: STDP, Synaptic Normalization (SN) of the excitatory-excitatory connections, and Intrinsic Plasticity (IP) regulating the thresholds of excitatory units. All plasticity rules here only apply to excitatory units and connections between excitatory units.

#### Spike timing dependent plasticity (STDP)

Is a temporally asymmetric way of adjusting the strength of connections between neurons. If the presynaptic neuron fires slightly before the postsynaptic one, the synapse between them will be strengthened. Conversely, if a postsynaptic neuron fires before the presynaptic one, the connection will be weakened:

$$\begin{array}{c} \Delta W_{ij}^{EE}\left(t\right)=\eta_{\text{STDP}}\left(x_{i}\left(t\right)x_{j}\left(t-1\right)-x_{i}\left(t-1\right)x_{j}\left(t\right)\right). \end{array}$$ (3)

#### Synaptic normalization

Prevents a weight from becoming infinitely large, and proportionally adjusts the values of incoming connections to a neuron so that they sum up to a constant value. Specifically, the *W*_*EE*_ connections are rescaled at every time step according to:

$$\begin{array}{c} W_{ij}^{EE}\left(t\right)\leftarrow W_{ij}^{EE}\left(t\right)/\sum_{j}W_{ij}^{EE}\left(t\right). \end{array}$$ (4)

#### Intrinsic plasticity

Ensures that on average each excitatory neuron will fire with the same predefined target rate *H*_IP_. At each step a neuron changes its threshold according to:

$$\begin{array}{c} T_{i}^{E}\left(t+1\right)=T_{i}^{E}\left(t\right)+\eta_{\text{IP}}\left(x_{i}\left(t\right)-H_{\text{IP}}\right). \end{array}$$ (5)

In spite of the constant synaptic modulations introduced by STDP, the two homeostatic mechanisms intrinsic plasticity and synaptic normalization along with the sparse connectivity between excitatory units ensure healthy network dynamics where the activity is asynchronous and irregular and the network exhibits good learning behavior [5].

#### Network parameters

**Fig 2:**
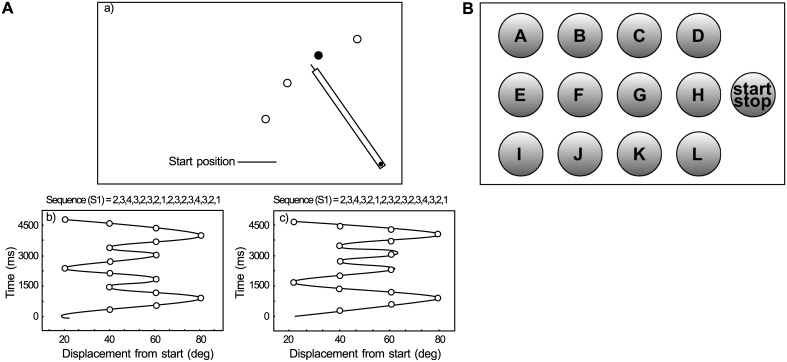
Experimental setups (A) Experimental setup of the Panzer et al. studies [24, 25]. a) Drawing illustrating the targets’ positions relative to the lever and start positions (Adapted from [24, 25]). The targets’ positions were projected on the tabletop from above. When the pointer attached to the manipulandum passed into the illuminated target, the illumination was turned off and the next target in the sequence was illuminated. Circles overlaid on the movement pattern indicate the position of the targets. The illumination of the target (filled circle) indicated the next target in the sequence. b) and c) are examples of the movement patterns produced for sequences 1 and 2. The time series examples in b) and c) were typical of patterns participants produced midway through the practice session. (Adapted from [24].) (B) Experimental setup of the Koedijker at. el experiments [26]. Schematic illustration of the arrangement of buttons utilized in the sequence learning experiments. (Adapted from [26]).

For all experiments, we used a network with *N*_E_ = 300 excitatory neurons and *N*_*I*_ = 60 inhibitory neurons. The maximum threshold values for the excitatory units was $T_{\text{max}}^{E}=0.5$ and for inhibitory units $T_{\text{max}}^{I}=0.9$. The connection probability between excitatory neurons was set to *p*_connect_ = 0.1, and the number of input neurons for a single element was set to *N*_input_ = 10. The learning rate of the two plasticity mechanisms were set as follows: learning rate of IP *η*_IP_ = 0.002, learning rate of STDP *η*_STDP_ = 10^−4^. In our simulations of the human sequence learning experiments (Fig 2), we used the SORN with three plasticity rules and compared it with the equivalent network in which the plasticity mechanisms were switched off.

#### Network output

We used a linear output layer that generates an output at every time step, thereby producing the movement towards the next predicted sequence element. We implemented a standard winner-take-all mechanism, i.e. the output neuron, which has the highest activity defines the output of the network at that time step. Thus the output of the network can be regarded as a movement that has to be carried out to reach the next predicted sequence element. The output weights, i.e. the connection strengths, between the reservoir and output neurons were trained so as to minimize the squared difference between the activity of the output neurons and the target output values. This was achieved by applying the Delta-rule [30] to the read out neurons’ weights at each time step of the simulation. The learning rate for the output weights was set to: *μ* = 2 × 10^−5^.

#### Network pattern separability and human performance modeling

The network activity needs to be related to the subjects’ behavioral performance. The SORN does not develop fixed point attractors but encodes spatio-temporal input patterns as spatio-temporal activity patterns. In doing so, it learns to predict the next sequence element. In fact, it has been shown previously that even when the input is removed, the SORN will spontaneously replay learned evoked activity patterns, which was subject of a recent study [23]. The quality of this internal prediction is directly related to how well the network manages to map different inputs onto distinct activity patterns. Therefore we chose a measure of the separation of different network states as a proxy for subjects’ reaction times, which also depend on the subject’s ability to correctly predict the next element. Separability here was defined as the sum of all pairwise distances between internal network activity patterns in response to an input sequence. The length of the activity patterns was chosen to be identical to the length of an input pattern, i.e. for a learning task involving input sequences of 20 elements corresponding to 20 time steps in the simulation, the length of the activity patterns was chosen to be 20. Thus, denoting with *X*_*t*_ the vector of length N collecting the activities of all network neurons in the reservoir excluding the input neurons at time *t*, we calculated the separability of network states at time t as: $S_{t}=\sum_{n=0^{19}}\sum_{m=0^{19}}∥X_{t+n}-X_{t+m}{∥}_{2}$.

### Methods for evaluating networks’ behavior

To understand the mechanisms underlying changes in the neuronal activities across learning and mediating the differences in generated motor sequence behavior we carried out a number of different analyses of network activities, which are detailed in the following section.

#### Separability and PCA of neuronal activities

In reservoir computing, performance depends on the separation property. A key requirement for good performance is that different inputs to the reservoir network result in separable internal states [31]. A larger separation in the reservoir’s state space means that it will be easier to classify different input streams. Various ways to measure separation have been studied, for example, in [27] the separation between two different network states is calculated by measuring the Euclidean distance. A similar geometric interpretation has been given by [32], which measures the separation of the network as the Euclidean distance between the centroids of the network states that belong to different classes. In [31], the authors use spike train distance metrics instead of the Euclidean distance. From the perspective of a classification system, it has been suggested that the rank of the state matrix, i.e. the matrix consisting of the concatenation of vectors of network activity vectors, can be used to measure the quality of the network [33]. According to this measure, the larger the number of linearly independent state vectors produced by a network state, the better the classification that can be obtained [34].

To better understand how plasticity mechanisms induce changes in the network underlying the performance improvements, we compare the SORN implementing three types of plasticity mechanisms to versions of SORN without STDP and IP mechanisms. We measure separability as the Euclidean distance between network states and also perform principal component analysis (PCA) on the networks’ internal state representations. At each time point, the vector of activities of the excitatory neurons in the reservoir forms n internal state representation of the network. The internal state representation was saved during training, and then PCA was performed on the saved internal state representations after training.

#### Agglomerative hierarchical clustering of neuronal activities

To obtain further indications of the representational changes in the network across training, we carried out agglomerative hierarchical clustering with vectors of networks’ internal state representations (*R*), as in [20]. Each pattern of activity *R*_*i*_(*t*) is a point in a space with dimensions of the size of the reservoir. Agglomerative clustering starts by considering each network activity vector and proceeds by successively joining the closest activity patterns into clusters. As distance metric we use the Euclidean distance between activity patterns. This process is repeated until all data are conjoined into a single cluster. The process of merging clusters can be stopped when a desired number of clusters is reached an in the present experiments we fixed the number of clusters to 20.

#### Evolution of the network’s excitatory weights

We analyzed the evolution of the network’s excitatory weights during training. In particular, we considered the incoming weight vectors into the excitatory units as points in a *N*_*E*_ dimensional “weight” space and followed their movement in this space. We define the vector of incoming excitatory weights into excitatory neuron *i* as $W_{i,*}^{EE}\equiv {\left(W_{i,1}^{EE},\ldots \;W_{i,N_{E}}^{EE}\right)}^{T}$. Specifically, we were interested in the question how switching from learning a first sequence to learning a second sequence affects the movement direction of these weight vectors. To visualize this movement in weight space, we also performed PCA on the set of all incoming weight vectors across the entire training and projected these weights into the lower-dimensional space spanned by the first three PCs.

#### Selectivity index

To further investigate changes within the network’s activity due to the plasticity mechanisms active in SORN, we analyzed neurons’ selectivity for different inputs. The selectivity index was introduced by Moody et al. [35] to quantify the degree of direction tuning for primary visual cortex cells. It quantifies whether a unit is firing strongly in response to all different conditions (in their case eight stimulus directions) versus in only one specific condition (one direction). The selectivity index of the *i*^*th*^ neuron was defined as follows:

$$\begin{array}{c} d_{i}=\frac{k-(\frac{\sum_{n=1}^{k}i_{n}}{i_{\text{max}}})}{k-1}, \end{array}$$ (6)

where *k* is the number of different input conditions; *i*_*n*_ is the neuron’s average firing rate responding to a target input *n*; *i*_max_ is the maximum response across all conditions. A value of *d*_*i*_ = 0 indicates no selectivity, i.e. that the neuron has identical responses to all stimuli; a value of *d*_*i*_ = 1 indicates high selectivity: the neuron is activated by one specific stimulus and does not respond to other stimuli [36].

#### Mutual information between input sequences and neuronal activities

Computing mutual information between the sequences and neuronal activities is a further way of quantifying how well the activities of model neurons in response to their input allow inferring the respective identity of the driving sequence. We can therefore ask: how much information about the stimulus is represented by the cell’s activity? We use Shannon’s mutual information and calculated from the joint probability distribution according to:

$$\begin{array}{c} I\left(R;S\right)=\sum_{r\in R}\sum_{s\in S}P\left(r,s\right)\log(\frac{P(r,s)}{P(r)P(s)}) \end{array}$$ (7)

In our case, the first variable *r* is each neuron’s activity (on or off), the second variable *s* represents different inputs to the network. *R* and *S* are the sets of values that *r* and *s* can take. *P*(*r*) is the overall probability that a neuron fires and *P*(*s*) is the probability of one input versus all input conditions. The joint probability *P*(*r*, *s*) is the probability that one neuron fires under a certain input condition. The ratio compares this joint probability to what might happen if firing were independent of the stimulus: the product of the two individual probabilities *P*(*r*)*P*(*s*). Finally, ∑_*r*∈*R*_∑_*s*∈*S*_ *P*(*r*, *s*) simply indicates taking a sum over all stimuli and all responses, weighted according to how often the combination occurs.

Numerically, we calculated the involved quantities as follows:

$$\begin{array}{c} P_{i}=\left(F_{\text{total}}/N_{\text{word}}\right)/L_{\text{word}} \end{array}$$ (8)

$$\begin{array}{c} Q_{i}=1-P_{i} \end{array}$$ (9)

$$\begin{array}{c} MI_{i}=\sum_{i\in N}\left(P_{i}\log\left(\frac{P_{i}}{\sum_{i\in N}P_{i}/L_{\text{word}}}\right)\right)+\sum_{i\in N}\left(Q_{i}\log\left(\frac{Q_{i}}{\sum_{i\in N}Q_{i}/L_{\text{word}}}\right)\right), \end{array}$$ (10)

where *P*_*i*_ is the overall probability that neuron*i* fires, *Q*_*i*_ is the overall probability that a neuron *i* is silent. *F*_total_ is total number of times neuron i fires during this block of training. *N*_word_ is the total number of input words in this block of training. *L*_word_ is the number of elements within each input word. In our training sessions, all input words have the same length.

## Results

### Modeling sequence-learning tasks

Overall, we carried out five different experiments to address sequence learning tasks in SORN and elucidating the connection between facilitation and interference effects on the one hand and task similarities and training schedule on the other hand. First, we made sure that SORN is able to reproduce some of the key aspects of previously published behavioral work with a single set of network parameters across all simulations. To this end we modeled human sequence learning tasks published in Panzer et al. [24], the tasks with altered learning times published in Panzer et al. [25], and a sequence learning task involving finger tapping published by Koedijker et al. [26]. Based on these results, we devised two sets of additional experiments addressing sequence element representations and investigating joint effects of task similarity and training schedule.

### Investigating facilitation and interference effects

#### Arm movement sequence learning tasks

Facilitation and interference effects are pervasive in human sequence learning and have long been described (see e.g. [1]). Panzer et al. [24, 25] carried out experiments to determine the extent to which the learning of one movement sequence influences the subsequent learning of a similar movement sequence. Participants produced sequences by moving a lever with their right arm and hand to sequentially presented target locations. They practiced two similar 16-element movement sequences (S1 and S2), with 14 of the 16 elements common in both sequences.

The fulcrum of the lever, which rotated freely in ball-bearing supports, allowed the lever to move in the horizontal plane over the table surface, as shown in Fig 2. The horizontal movement of the lever was monitored at 200 Hz by a potentiometer that was attached to the lower end of the axle. The potentiometer data were used to provide lever position information to the participant and stored for later analysis. The targets and total movement time were projected on the table surface by a projector mounted above.

To encode the direction of a movement, we define the upward movement e.g. from position 2 to 3 with the symbol 2, differently from the downward movement e.g. from position 3 to 2 with symbol 3’. Thus, position and direction of movements are encoded jointly and result from activations of different subgroups of input neurons in SORN. We also add four extra elements (A1-A4 vs. B1-B4) in the beginning of the sequences corresponding to the lead-in movement [37], which constitutes a signal for detecting contextual differences between S1 and S2. In the simulation of the experimental group, we ran the SORN for 8000 time steps to learn S1 and 8000 time steps to learn S2. In the control group, only sequence 1 or sequence 2 was trained.

In the simulation we use sequences with length 20:

Sequence 1 (S1) = [A1 A2 A3 A4 2 3 4 3’ 2’ 3 2’ 1’ 2 3 2’ 3 4 3’ 2’ 1’], and
Sequence 2 (S2) = [B1 B2 B3 B4 2 3 4 3’ 2’ 1’ 2 3 2’ 3 2’ 3 4 3’ 2’ 1’].

Each digit in the sequence corresponds to a target position in the psychophysical experiment in Fig 2, and we call it an ‘element’of the input sequence in our simulation. Each element in the input sequence corresponds to external input to a subset of 10 input neurons. There is no overlap of activity of the input neurons between different input elements. Input neurons are connected to the excitatory reservoir neurons as shown in Fig 1.

#### Facilitation and interference effects in arm movement sequence learning tasks

In Experiment 1 [24], participants were split into 3 groups, one experimental group and 2 control groups. The experimental group practiced two movement sequences, one sequence on each of two consecutive days of practice. The Sequence 1 (S1) with 16 elements was practiced on Day 1 and Sequence 2 (S2) on Day 2, where S2 was created by switching 2 positions of 16 elements in sequence S1. The control groups received only one day of practice on one of the sequences. Control group 1 only practiced S1 on Day 1 and Control group 2 only practiced S2 on Day 2. On Day 3, all groups were tested on both S1 and S2, participants were counterbalanced in order.

**Fig 3:**
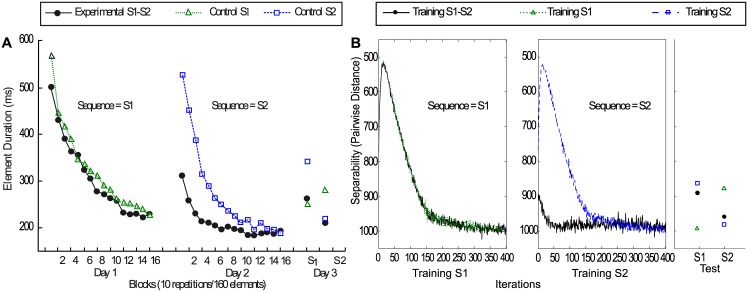
(A) Results of Experiment 1 [24] (Adapted from [24]). Acquisition, retention and transfer performance of the experimental and control groups. Note that Sequence 1 (S1) was practiced on Day 1 and Sequence 2 (S2) on Day 2. On Day 3, all groups were tested on both S1 and S2, in counterbalanced order. (B) Simulation of experimental and control groups. Sum of pairwise distance of network states under different input conditions using Euclidean norm. Circles: experimental group, training S1 and then S2 with equivalent training time. Triangles: control, training on S1 only. Squares: control, training on S2 only. Proactive facilitation was observed in the beginning of training S2, comparable to the psychophysical data, as performance of the network simulating the experimental group is better compared to the control group training. When training time is equivalent for S1 and S2, the performance of the network also shows retroactive interference. The tested separability of the later sequence (S2) is larger, implying that the second sequence has been learnt better [24]).

Experiment 1 results are shown in the left part of Fig 3. Early in Day 2 (S2) practice, the experimental group demonstrated a relatively strong level of proactive facilitation arising from previous practice with S1, which is due to the high similarity between sequences S1 and S2, which have 14 of the 16 elements in common. On Day 3, the experimental group showed a strong retroactive interference on the switched elements. The tested performance on S2 was better compared with S1, and the tested performance on S1 was worse compared to the performance at the end of Day 1. Thus, the memories underpinning S1 seemed to be overwritten or adapted in response to the learning of S2.

As the result, in Fig 3 the black circles represent the experimental group, in which the network first was trained on S1 and consecutively on S2 with the same training time (400 data points, each of which contains 20 time steps). In the control conditions (triangles and squares), the network was trained with only one sequence with the same training length. Referring to the simulation results displayed in Fig 3, early in S2 training of the experimental group (black circles), the performance was distinctly better than the starting performance for S1, which indicates facilitation from the previous training of S1. In the test phase for the experimental group, the performance on the later sequence (S2) was better compared to that for S1, which is consistent with the retroactive interference observed in the psychophysics experiment. Thus, the SORN reproduces the interference and facilitation effects that were observed in the human sequence learning experiments. We were interested to obtain an indication of the robustness of our results to the variation of networks’ parameters. To this end, we varied two of the essential parameters that describe the network, namely the sparsity of the connections between excitatory neurons governed by the *p*_*connect*_ and the ratio between the number of excitatory neurons *N*_*E*_ and the number of inhibitory neurons *N*_*I*_. The results of these experiments are reported in the supplementary material. These results show, that the pattern of performance for the sequence learning tasks is maintained over values for *p*_*connect*_ ranging from a value of 0.05 to 0.15 and that similarly the performance of the network is maintained for a ratio of inhibitory to excitatory units between 0.1 and 0.4.

#### Facilitation and interference effects in arm movement sequence learning tasks with altered training durations

**Fig 4:**
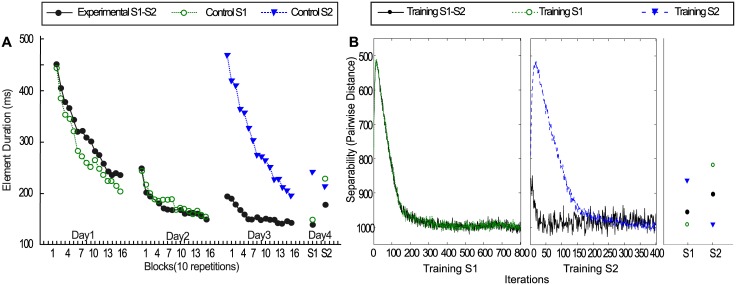
(A) Experiment results of experiment 2. (Adapted from [25]). Acquisition, retention and transfer performance for the experimental and control groups. Note the experimental group practiced S1 on Days 1 and 2 and S2 on Day 3. The S1 control group practiced S1 on Days 1 and 2. The S2 group only practiced S2 for one day. All groups were tested on S1 and S2 (order counter-balanced). (B) Simulation results for [25]. Same as above, with the difference that training on S1 is twice as long. Note that now the retroactive interference is attenuated, as in the psychophysical experiments.

Experiment 2 [25] was carried out originally to investigate how prolonged practice on a sequence would influence performance on the sequence learning tasks previously described as experiment 1 [24]. In Experiment 2, the same two 16-element movement sequences (S1 and S2) were used as in Experiment 1. Experiment and simulation results are shown in Fig 4. The experimental group practiced the first sequence (S1) for two consecutive days (Day1 and Day2), which is twice as long as the previous experiment. A second sequence (S2) was practiced on Day 3. Control groups received either two days of practice on S1 or one day of practice on S2. The proactive facilitation in the early stage of S2 acquisition was observed as in the previous experiment. Contrary to the earlier findings in Experiment 1 [24] of strong retroactive interference when S1 was only practiced for one day, this time no evidence of retroactive interference was found when S1 was practiced for two days. When S1 was tested on Day 4, the performance of S1 was about the same as it was tested after the first two days of training (Day 1 and Day 2), and the performance of S1 was better compared with S2.

Based on the previous simulation experiments, we adapted the training schedule to reflect the psychophysical manipulations. In the simulation of Experiment 2 the experimental group (black circles in Fig 4) was trained twice as long on S1 as on S2. In Fig 4, the left panel shows the training of S1, 20 × 800 = 16000 time points were analyzed and displayed, the middle panel shows the training of S2 (20 × 400 = 8000 time points). The third panel shows the result of testing S1 and S2.

The simulation results are displayed in Fig 4. The solid black curve shows that the tested performance on S1 was about the same as the performance at the end of training S1, consistent with the psychophysics experiment with no retroactive interference. A possible explanation is that the longer training time on S1 lead to changes in the network such that the dynamics in response to S1 are more stable compared to the shorter training on S2 and more training time on S2 would be needed to result in an interference effect on S1. Inspecting the performance of the network after training (see third panel of Fig 4B) shows the same relative performance on S1 versus S2 as in the psychophysical experiments across all training groups, i.e. training on S1 and subsequently on S2, training only on S1, and training only on S2. However, there is one difference to the simulations of the network, as the experimental S1-S2 group performs better at testing than the control S2 group. Note however, that both the training and testing schedule for the control group S2 are identical in both experiments [24] and [25], but human performance at testing is different in the two studies. While the reported results of the first study [24] match the simulations quite well, we attribute the deviations in the second experiment to variability inherent in the experimental data with human subjects. A detailed analysis of the variability in human performance as well as further work on the variability in the simulations could clarify the source of this deviation.

In this section we have shown that a single network using only a single set of parameters can display performance that mimics the performance measures observed in human subjects for the considered psychophysical tasks. The following section presents a more detailed analysis of the underlying changes in the neuronal activities generating motor sequences across learning.

#### Network separability analysis

**Fig 5:**
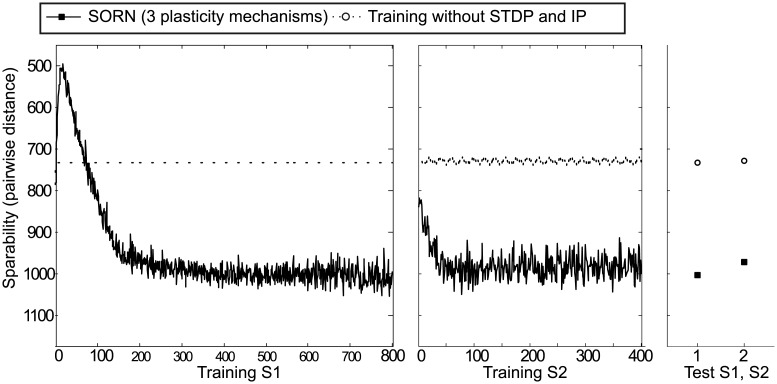
Seperability of SORN with all three plasticity mechanisms turned on (solid) or with STDP and IP turned off (dotted). To be intuitively comparable to experimental data, the Y-axis is plotted upside-down.

**Fig 6:**
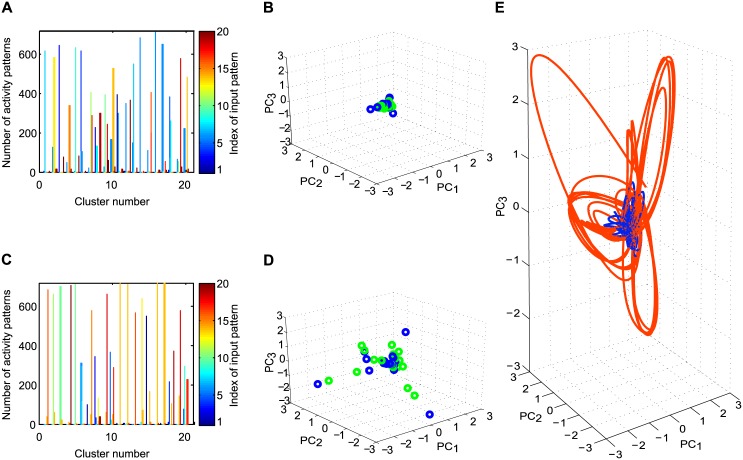
(A) Result of clustering of the internal activity patterns of the reservoir in the beginning of training. Clusters tend to mix many distinct input conditions, and mix different repetitions of the same input, instead of keeping them separate. (B) Projections of initial network activities into the space spanned by the first three principal components of activities throughout learning. Individual points corresponding to neural activity patterns in response to individual elements of the first sequence S1 (blue) and to individual elements of the second sequence S2 (green) are lumped closely together at the beginning of training. (C) The cluster structure after training the SORNs shows that activity patterns are more distinct among the 20 clusters and that a smaller number of differing patterns are assigned to individual clusters. (D) Projections of final network activities into the space spanned by the first three principal components of activities throughout learning. Individual points corresponding to neural activity patterns in response to individual elements of the first sequence S1 (blue) and to individual elements of the second sequence S2 (green) are lumped closely together at the beginning of training. After training, the network states are distributed in the shape of a tetrahedron. The volume covered by these states has increased in comparison to the beginning of training. Note that the two sequences used in these simulations had a task similarity of 0.25. (E) Trajectories of network activities in the space of the first three PCs. The network activity in the low dimensional embedding space shows that the separation of activities in the network grows across training from the initial 20 training elements (blue) to the final 20 elements (orange). The trajectory of activities was plotted using cubic spline interpolation in the low dimensional PC space of network activities across time for illustration purposes.

To gain insight into the changes occurring within SORN across learning we trained networks with and without plasticity mechanisms and computed the separability of internal network activities for these differently trained networks. Indeed, adapting the network using the full set of plasticity mechanisms improves performance in the initially random circuit and greatly increases separability compared to the initial random network, as demonstrated in Fig 5. The SORN network with all three forms of plasticity is plotted in dark solid lines in training and dark solid squares in testing. Network without STDP and IP plasticity is plotted in dotted lines in training and empty circles in testing. This provides clear evidence, that the results of modeling the motor sequence learning tasks in the previous sections crucially depended on the plasticity mechanisms in SORN. A visual illustration of the separability of spatio-temporal network activities can be obtained by plotting the sequence of network states in a low dimensional space obtained through the first three principal components of the PCA, as in Fig 6B and 6D. Thus, the full set of plasticity plasticity mechanisms improves the randomly initialized circuit and greatly increases separability.

#### Agglomerative clustering of network activities

The results of agglomerative clustering are shown in Fig 6A and 6C, where different colors reflect the respective clusters. For this clustering, higher peaks reflect better performance, meaning that a cluster encodes different trials of the same input. The number of peaks should be minimized such that, in an ideal case, each cluster would have one peak meaning that it represents only one input pattern. Inspecting these plots shows that in the beginning of training, the evoked network responses to different inputs can be quite similar and fall within the same cluster. Thus many different input conditions contribute to a single cluster of network states (Fig 6A). For each of the 20 clusters, a histogram depicts the counts of neuronal activity patterns in response to the input sequences that contributed to the cluster, with averagely 3.75 different input conditions contribute to each cluster, with each input condition contributing to 34.58%. Thus, clusters tend to mix many distinct input conditions, and mix different repetitions of the same input, indicating no clear separation between the inputs. By contrast, in Fig 6C, there are fewer short bars, and instead more long bars for each cluster, indicating that plasticity separated the internal representations. With each cluster containing average 1.7 input conditions, with average 71.67% from each cluster in Fig 6C. Inspecting the expanding separation in PCA space, in the beginning of the training, input conditions produce a cloud of network states that substantially overlap with those from other input states within the projection space of the first three principal components (PC), as shown in Fig 6B. By contrast, after training the SORN has developed an internal representation where input conditions produce clusters of network states that are well separated from those of other input conditions (Fig 6D). When we compare the space spanned by the dots in Fig 6D versus 6B, the SORN develops an internal representation where input conditions produce tight clusters of network states, and the separation in PCA space after training is larger than before training. Furthermore, the first three PCs in the SORN capture a greater amount of variance compared to random networks. In particular, SORN learns to distinguish different states that have a very similar history of inputs, say, repetitions of the same input condition. This leads to more orderly and stereotyped trajectories through the network state space in the case of SORNs. This is also reflected in a greater amount of total variance of network activity which is captured by the first few PCs in the SORN when compared to random networks (not displayed here).

#### Evolution of the network’s excitatory weights

**Fig 7:**
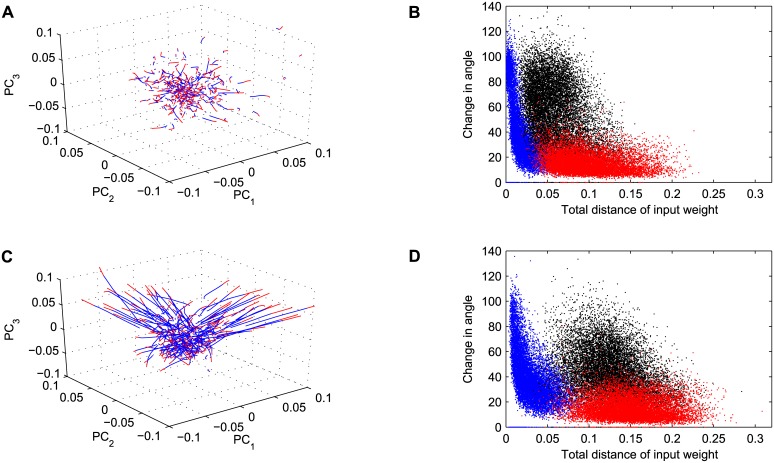
Analysis of changes in input weight vectors over training. (A) Euclidean distance of all 300 weight vectors from the initial weight $W_{i,*}^{EE}\left(0\right)$ across training in Experiment 1 [24] projected into the space of the first three PCs of all weight vectors. The blue part of each curves corresponds to training on sequence S1 and the red part corresponds to training on sequence S2. (B) Change in angle of the weights versus the total distance of the weights to the initial weight across training in Experiment 1. The angle was computed through the dot product in the full 300 dimensional space of weights. The total distance of weights was computed as the Euclidean distance of the weight vectors $W_{i,*}^{EE}\left(t\right)$ to the initial weight vector $W_{i,*}^{EE}\left(0\right)$. The blue data points correspond to the respective values after the first 1600 training steps and the red data points correspond to the weights after the last training step. The black data points correspond to the changes in angle versus distance in weights across training after switching from sequence S1 to sequence S2. Panels (C,D) as Panel (A, B) but for the data of Experiment 2 [25].

To shed light on the mechanisms responsible for the qualitative difference between long and short training durations, we analyzed the evolution of the network’s excitatory weights during training. Fig 7A shows an example of the movement of each of the 300 weight vectors during training for short S1 training duration before switching to S2, with the origin representing the initial weight vector $W_{i,*}^{EE}\left(0\right)$ at *t* = 0. For visualization purposes we projected weight vectors in the lower-dimensional space spanned by the first three principal components of all weight vectors across the entire training.

The blue part of each trajectory corresponds to training on sequence S1 and the red part of each trajectory represents the subsequent period of training on sequence S2. First, we observed that there is a general trend for the weight vectors to move outwards from the origin during training of S1. This movement is associated with an increasing number of individual weights that go all the way to zero. At the time of switching to S2, many weight vectors change direction, reflecting the network’s adaptation to the new input sequence. Interestingly, however, these changes in direction are more pronounced for weight vectors that are still close to the origin. This effect is illustrated in Fig 7B, which shows a scatter plot of the change in angle of the weight vectors in the full 300-dimensional space as a function of their distance from the origin at three different time points. The difference in angle is calculated over 1600 time steps: blue points represent the change in direction of the weight vectors on the first training step, red for the last training step, and black points represent the change of angle at the time when training switched from S1 to S2.

Several findings are apparent: First, there is a general trend for weight vectors that are far from the origin to undergo smaller changes in direction (compare blue and red populations). Second, at the time of switching from S1 to S2, large changes in direction are observed, but they tend to be smaller for weight vectors that have already moved far from the origin.

Fig 7C shows the movement of weights in PC-space for long training duration of S1. Note that during the extended training time of S1 the weights move further away from the origin (blue parts of the trajectories). When training switches to S2, most of the weight vectors that have moved far away from the origin during S1 training barely change direction but continue to move in the same direction. Fig 7D shows a scatter plot of changes in angle vs. distance from the center for long S1 training duration. Compared to Fig 7B, the longer S1 training has led to weight vectors with greater distance from the center (compare blue populations in B and D). Furthermore, upon the switch to S2 changes in direction of the weight vectors tend to be somewhat smaller than for short S1 training (compare black populations in B and D). To quantify the effect, we directly compared the changes in direction of the weight vectors at the time of the switch from S1 to S2. For short S1 training duration the average change in direction in the full 300-dim. weight space was 57 ± 24 degrees, but it was only 44 ± 19 degrees after long S1 training. This difference was highly significant (t-test, *p* < 10^−32^).

These results suggest the following explanation for the difference between short and long S1 training durations in Figs 3 and 4. With increasing training time for S1, the memory trace of S1 becomes more deeply engraved into the network’s structure. The longer this process lasts, the harder it becomes for the network to learn a new sequence which requires a change in the direction of the network’s weight vectors. The reason for this is that the activity patterns of the SORN are a product of both the external input and the already established recurrent connectivity. As training on S1 progresses the network weights come to more distinctly reflect the spatio-temporal structure of S1 and the network becomes less sensitive to changes in the input structure. This is because the recurrent connectivity gains a growing influence over the network’s activity patterns, which ultimately drives changes in the network structure via the STDP rule. In fact, previous work with the SORN has already shown that after sufficiently long training, the network’s activity will be a product of both external inputs and its learned recurrent connectivity. Furthermore, in the absence of any external input, the network will spontaneously replay learned sequences [23]. This illustrates the strong influence of the recurrent connectivity on the network’s activity and therefore also on further changes to its weights.

#### Selectivity index

**Fig 8:**
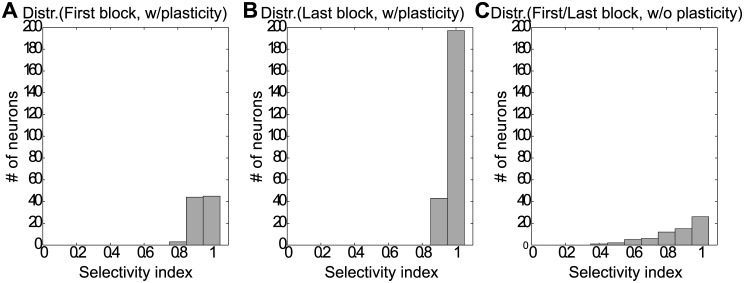
Selectivity indices of neurons in SORN network. (A) Distribution of selectivity indices in SORN with plasticity in the first block of training. (B) Distribution of selectivity indices in SORN with plasticity after training (last block), which becomes higher with training compared with the first block. (C) Distribution of selectivity indices in SORN without STDP and IP plasticity. A value of zero indicates that the neuron has identical responses to all stimuli; a value of 1 indicates activation by one stimulus and silence to all other stimuli. In SORN, neurons are firing highly selectively, with the indices reaching values between 0.9 and 1.0.

We compared the selectivity indices of all neurons in the reservoir for the SORN after the first block of training and after training was completed to the selectivity indices in the network without plasticity. The resulting histograms of the selectivity indices are depicted in Fig 8. When the plasticity mechanisms are switched off, the connectivity between neurons in the reservoir is not changing and the selectivity is determined by the random connectivity present in the network from the beginning. In this case, the number of neurons with high specificity is limited and across the network selectivity indices can be as low as of 0.4 (Fig 8C). By contrast, the plasticity in the SORN changes the connectivity of the network in such a way that the neurons become more selectively tuned to the inputs. Already after the first block of training, plasticity has shaped the selectivity so that the index for most cells lies between values of 0.9 and 1 (Fig 8B). After the last training block, the vast majority of units in the SORN have become highly specific to the input and show a selectivity index close to 1(Fig 8B).

#### Mutual information analysis

**Fig 9:**
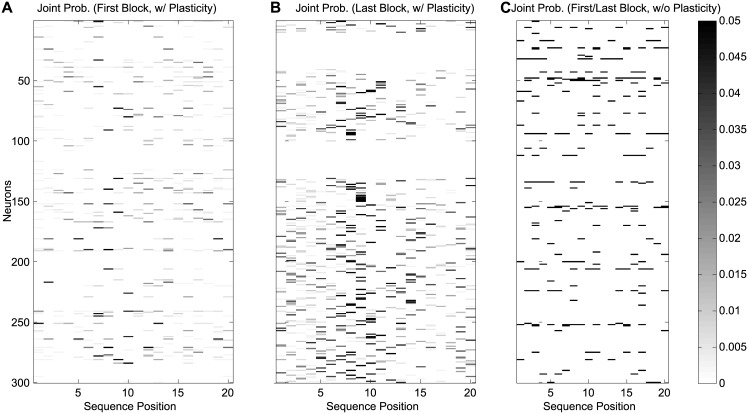
The joint probability between inputs and neuron firing. The vertical axis corresponds to the network’s 300 model neurons, and the horizontal axis corresponds to the input sequence element of the total length 20. (A) Joint probability of inputs and neuron firing in SORN with plasticity in the first block of training. (B) Joint probability of inputs and neuron firing in SORN with plasticity in the last block of training. Compared with the left subplot, the joint probability becomes higher with training and the firing of neurons is sparse. (C) Joint probability of inputs and neuron firing in SORN without STDP and IP.

We plotted the joint probability between input sequence and the neuron firing in Fig 9. In this figure, the vertical axis shows individual neurons (300 neurons), and the horizontal axis represents the 20 different inputs, with some elements appearing more than once in the input sequence. Every 20 trials (400 input elements) was defined as one block, we plotted the first block and the last block of training in Fig 9. Comparing subplot A and B, one can see the increase in the joint probability of firing throughout training of the SORN network as reflected by initial low probabilities (represented by lighter shades of gray) to higher joint probabilities (represented by darker shades of gray). The correspondences between the neurons and input positions are very sparse, which gives different inputs different firing patterns in the network, so different inputs are distinguishable by the neurons’ firing patterns. Even when the input elements are the same, their representation of firing patterns are different across time. By contrast, when training the network without STDP and IP plasticity mechanisms, only very few neurons are activated overall.

**Fig 10:**
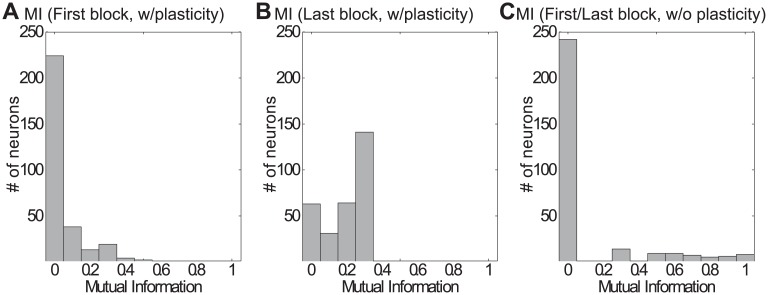
Mutual information distribution. A) Distribution of mutual information in SORN with plasticity in the first block of training. B) Distribution of mutual information in SORN with plasticity after training (last block), which becomes higher with training compared with the first block. C) Distribution of mutual information in SORN without STDP and IP plasticity.

Mutual information between inputs and the neurons’ response is plotted in Fig 10, with the left and middle subplot showing how mutual information will increase and how more neurons are activated across training with STDP and IP plasticity. The right subplot by contrast shows mutual information after training in the network without STDP and IP mechanisms. Only very few neurons show high mutual information with respect to the input sequences. This is very different from the SORN, in which more neurons show a non-zero mutual information value after training. The mean value of mutual information in the first block of training in SORN is 0.05, while the mean value of mutual information in the last block of training is SORN is 0.19, which is higher than the mutual information in the same network but without plasticity 0.12.

By definition, only neurons firing selectively to different inputs have large mutual information. In SORN with plasticity, a subgroup of input neurons (neuron number 21-40 and 121-140) became inactive, and had low joint probability and mutual information compared to other neurons (Figs 9B and 10B). These input neurons were assigned to frequently appearing inputs and in the beginning of training they were firing very frequently. After long training, the firing thresholds of the frequently firing neurons increased because of the effect of intrinsic plasticity and, once their firing threshold became too high, these neurons entered phases of inactivity.

### Task similarity and element analysis

From the considered experiments we can conclude that learning a sequence S1 can lead to proactive facilitation when subsequently learning a similar sequence S2. But arguably, this may not be the case for arbitrary sequences S1 and S2. In the considered cases, the sequences had a high similarity in terms of the overlap of positions and directions within the movement sequences. However, the measure in performance considers the whole task sequence. To study how task similarity can influence learning performance, for example, whether changes on specific positions will influence the learning locally, we carried out a position specific analysis of input sequences. Based on these results, we trained SORN on the discretized button press experiments by Koedijker at. el [26], in which subjects learned two tasks consisting of consecutively pressing eight target buttons in sequence. The two sequences, which had to be learned, differed on positions 4 and 5 within the sequences, which were exchanged.

#### Finger tapping sequence learning task

Koedijker at. el [26], conducted a discretized button press experiment as illustrated in Fig 2B. One sequence consisted of subsequently pressing buttons I-D-F-B-K-H-A-L, whereas the other sequence consisted of pressing buttons I-D-F-J-C-H-A-L. Both sequences consisted of consecutively pressing eight target buttons resulting in eight consecutive movements, with the first movement being the movement from the start button to the first target button (Button 1), the second movement being the movement from Button 1 to the second target button (Button 2), and so on, up to the movement from Buttons 7 to 8. The sequences differed on two buttons, that is, Buttons 4 and 5. The two changed buttons were mirrored to keep the between-button distances equal over the sequences.

**Fig 11:**
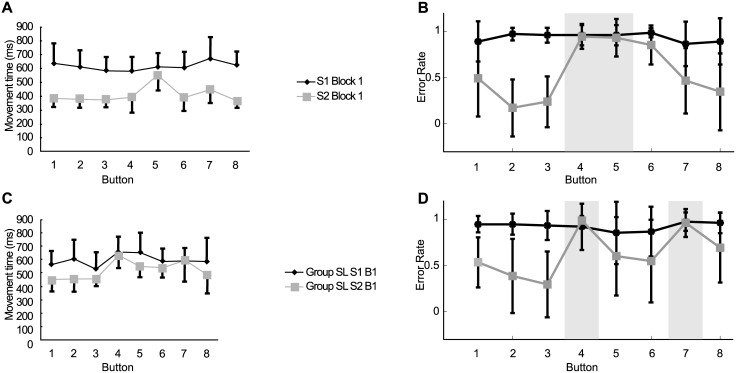
(A) Empirical data with changed Button 4, 5 (Adapted from [26]). Proactive facilitative effects are specific to the ordinal position within the learned sequence. (B) Simulation results of the experiment in A. (C) Empirical data with changed Button 4, 7 (Adapted from [26]). Position specific facilitation was observed. (D) Simulation results of the experiment in C).

Averages and standard deviations of reaction times across participants of both Sequence 1 (S1) and Sequence 2 (S2) of this Experiment are shown in Fig 11B to illustrate the short-term proactive facilitation effects. Button press times were averaged over the five recorded trials for each block. To test for proactive effects for both sequences the button press times over the six acquisition blocks were compared. Post hoc comparisons on the Sequence × Block × Button interaction indicated that on Block 1 all button times, except for Button 5, were significantly faster on S2 than S1. This tendency of Button 5 to show no facilitative effect on learning S2 was repeated on the following blocks. Thus, the proactive facilitation effect did not apply equally to all buttons, implying that the proactive effects were button-specific.

We again used the SORN with the parameter setting of the previous experiments to simulate these sequence learning experiments and our simulation data are shown on the right side (Fig 11C). Compared with the experimental data, not only Button 5 was influenced, but Button 4 and 6 as well. We argue that in the experiment only the reaction time was recorded but not the error rate, it might be the case that Button 4 and 6 are still pressed as fast as the unchanged buttons, but in fact with higher error rate. The sequences differed on two buttons 4 and 5, but the movements were changed to Buttons 4, 5, and 6. Although in the movement to Button 6 the target button remained the same, the button from which the movement had to be initiated was different. In our simulation we can see that the proactive facilitation effect did not apply equally to all buttons, but only to the positions for which movement was unchanged, implying that the proactive effects were dependent on specific position similarity, very similar to the effects observed in the psychophysical experiment.

An additional sequence was used in [26] to extend the findings of the above experiment by changing movements to Buttons 4 and 7 between the sequences. By altering the sequence we avoided capitalizing on effects that might have been specific to a certain set of movements within a particular sequence. The sequences consisted of sequential movements to button locations I-D-F-C-E-G-J-H and I-D-F-K-E-G-B-H. In the experimental data, averages and standard deviations of movement times across participants of Block 1 of both Sequence 1 (S1) and Sequence 2 (S2) are shown in Fig 11D. Pairwise comparisons revealed that all button times were faster for S2 compared to S1 (all *p* < 0.01), except movements to Buttons 4 and 7. Buttons 4 and 7 were the buttons of S2 that were different from the corresponding parts of S1. Thus, the results demonstrate a button-specific proactive facilitative effect for the movements that remained unchanged from S1 to S2, but not for the two buttons that were different from S1 to S2. The results from our network in the corresponding simulations are shown in Fig 11E. The proactive facilitation was observed for all positions except the changed buttons 4 and 7 just as shown in the experimental data. This demonstrates once more that the proactive effects were dependent on specific position similarity.

### Joint effects of task similarity and training schedule

In this section, we trained SORN with the same network parameters in all previous experiments on a large number of different sequence learning tasks to investigate the effects of task similarities and training schedules. We jointly varied task similarities between sequences, as quantified by the fraction of overlapping sequence elements, and training schedules, as measured by the number of blocks of training in which the training sequence is not altered. We show how task similarity and training schedule interact to produce a rich set of interference and facilitation effects thereby unifying procedural memory consolidation and structure learning in a recurrent network model with multiple plasticity mechanisms. This provides an implementational explanation of a rich set of behavioral phenomena as well as testable predictions for further experiments.

#### Training schedule: Blocked vs. interleaved

**Fig 12:**
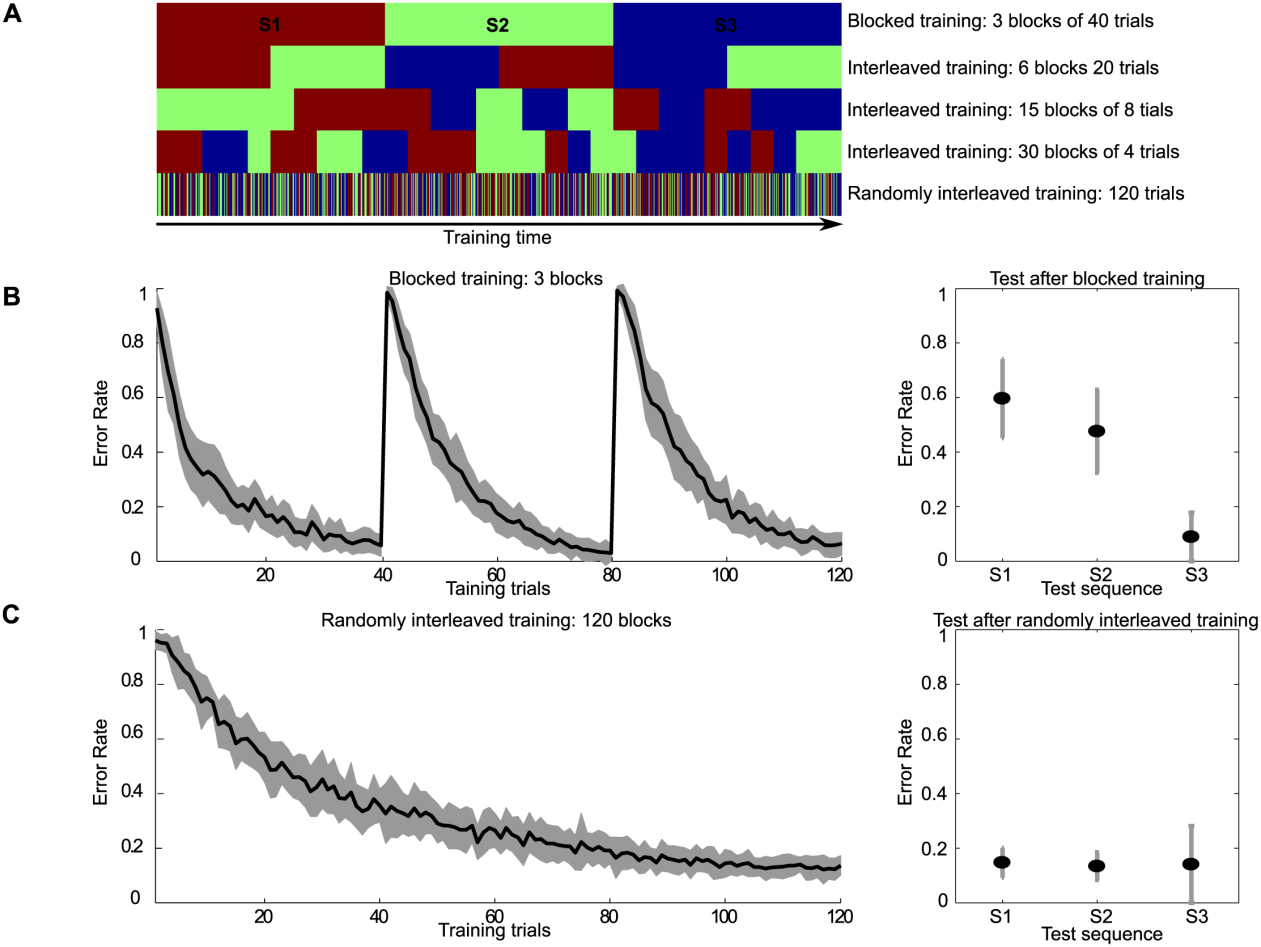
From blocked to interleaved practice. Different color stands for different tasks. (A) Illustration of different training schedules from top to bottom with 200, 100, 40, 20, and 1 trials per block. Together with a sequence length of 8 elements per trial this resulted in a total of 1600, 800, 320, 160, and 8 sequence elements per block, respectively. Each task is trained for the same total number of trails, and the blocks’ sequences were randomly generated. Therefore the upper training schedule corresponds to blocked practice while the bottom training schedule corresponds to randomly interleaved practice. (B) Blocked learning produces proactive interference, only the last task will have good performance in the end. (C) Randomly interleaved practice allows good performance in all three tasks after training.

An exciting possibility is that the stability of a memory trace is related to the training schedule. As show in Fig 12, with the same amount of total training time, short and interleaved practice sessions might produce a stable memory trace that is not susceptible to interference. But prolonged practice blocks might also generate less stable memory traces that show interference between tasks [1]. Indeed, Osu et al. [38] found that human subjects can adapt to two opposing force fields when provided with contextual cues and can consolidate motor memories if the force fields are interleaved in a random fashion. This study suggests that multiple internal models can be acquired simultaneously during learning and predictively switched, depending only on a contextual cue. By contrast, the literature on motor learning contains reports from experiments showing that if different tasks alternate frequently or are presented in large alternating blocks as in [39, 40], then learning of the second task can lead to an unlearning of the internal model for the first. There might also be potential benefits of interleaved practice when acquiring multiple finger movement sequences.

We use our network with the same parameter settings as before to investigate the differences between training schedule as illustrated in Fig 12. This investigation compares the performance of the network on three sequence learning tasks after using different training schedules. As shown in Fig 12A, the whole duration of the experiment can be subdivided in many different ways, e.g. in three consecutive blocks of uninterrupted training of each task, or in six consecutive blocks alternating between the three tasks, or in the limit, alternating between tasks on every individual trial. For the experiments we used three different sequences of length 8, where no sequence shared a common element with another sequence. As shown in Fig 12A we divided the whole training time (4800 time steps) into three blocks (with 1600 time steps in each block), each task was assigned to each block with one after the other, and trained with the same length of time (task S1 on block 1, task S2 on block 2 and task S3 on block 3). Different task performance was only measured after all training was completed. This testing after learning was carried out on the three sequences (S1 to S3) used during learning. In the lower panel of Fig 12A the whole training time was the same as in the previous one, and the three tasks were the ones used in the upper panel as well, however, the three tasks were randomly arranged during training with equal training time for each task.

The left panels of B and C in Fig 12 show the results of training of the three sequences after training, again, as in the figures reporting the results of the previous experiments. From the test results we can see that in the case of randomly interleaved training, all three tasks were performed well with a low error rate. However in the blocked training case, only the task trained last (S3) had good performance and the previous tasks (S1 and S2) showed signs of retroactive interference. These simulation results are consistent with experiments [38–40].

#### Interaction between task similarity and training schedule

In the previous section “Training schedule: blocked vs. interleaved” we could show the influence of blocked versus interleaved training schedules on the performance of the SORN in sequence learning tasks. Referring back to the results on position specific similarity between sequences, it is now natural to investigate how performance depends on task similarity and training schedule, if both are varied. We carried out learning experiments by jointly varying both task similarity and training schedule. The setting for the following experiments is identical as in the previous Section, i.e. the network was trained on three different tasks where each task was trained for 200 trials resulting in a total training duration of 600 trials and a sequence lengths of 8 positions for all three tasks. The training schedule was varied as described in Section. By varying the number of trials a single task was trained on consecutively. A blocked training schedule is achieved by training the three tasks for 200 consecutive trials in a total of 3 blocks whereas a randomly interleaved training schedule is achieved by switching the trained task randomly after every trial resulting in a total of 600 blocks. Intermediate training schedules were achieved using an intermediate number of of blocks. The full set of the number of blocks was {3, 6, 12, 24, 50, 100, 200, 400, 600}.

To simulate different task similarities, we need to choose a measure of sequence similarity. While a general measure of task similarity in sequence learning is not available, a commonly used measure to compare the similarity of sequences is the Hamming distance, i.e. the number of exactly matching inputs at the corresponding positions in two sequences. Here we used the complement of the Hamming distance normalized by the sequence length. Thus, a task similarity of 0 corresponds to no shared input among two sequences (for example, ABCD vs. EDFG vs. HIJK) whereas a task similarity of 0.25 could be ABCD vs. AEDF vs. AGHI, and a task similarity of 1 means that the 3 tasks are identical on every position of the sequence of the inputs (ABCD vs. ABCD vs. ABCD). For the present simulations we used eight equally spaced task similarities linearly increasing by 0.125 between 0 and 0.875.

Finally, to quantify the amount of anterograde and retorgrade performance effects across all training schedules and all tasks similarities, we needed to adopt a specific measure of interference and facilitation. Retrograde effects are commonly measured as the difference between performance at testing after the training of all tasks has been completed and performance at the end of training of that task, which is being considered [24, 25]. As an example, referring back to the experiments in Section., performance on sequence S1 at testing after blocked training had been completed was worse than at the end of training S1, constituting retrograde interference, as depicted in Fig 12. Accordingly, retrograde effects on S1 were quantified as the difference in error rates after the end of training S1 and the error rate at testing, when all training had been completed, averaged over the last five trials to reduce variability. Anterograde effects are quantified differently in the literature on sequence learning and for the following experiments we chose the difference in performance at the beginning of training two different sequences. Thus, if performance at the beginning of training sequence S2 is better than performance at the beginning of training sequence S1, then this constitutes anterograde facilitation, because having already trained on sequence S1 facilitates learning of sequence S2. Accordingly, anterograde effects on S1 were quantified as the difference in error rates at the beginning of training sequence S2, again averaged over five trials to compensate for variability in individual trials. Each combination of training schedule and task similarity was simulated with 40 randomly generated sequences of tasks and the averaged performance over the 40 experiments was separately computed for anterograde and retrograde effects.

**Fig 13:**
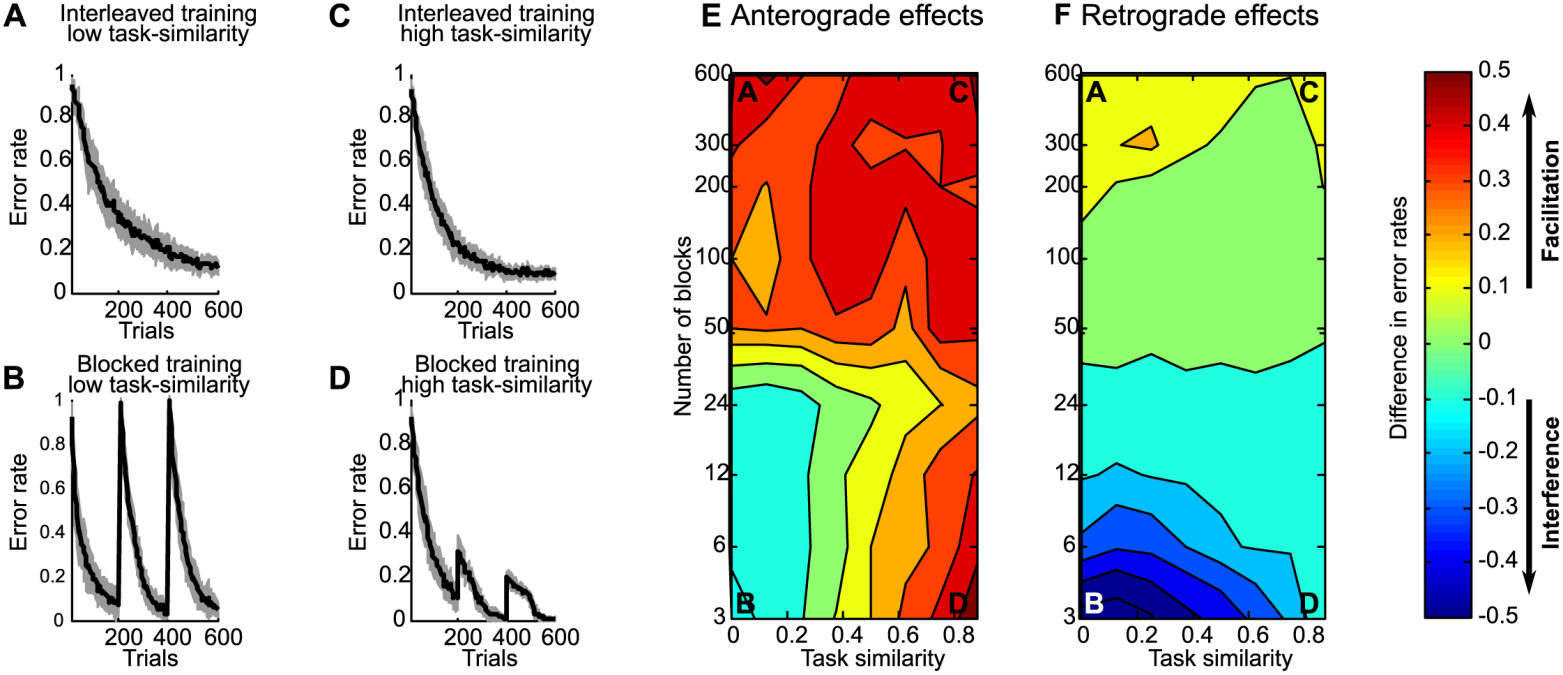
Anterograde and retrograde facilitation and interference effects across task similiarities and training schedules. A) Network performance as a function of the number of training trials for interleaved training with low task similarity. B) Performance for blocked training with low task similarity. C) Performance for interleaved training with high task similarity. D) Performance for blocked training with high task similarity. E) Anterograde effects in learning as quantified by the difference in error rates between the first 10 training trials on task S2 and the first 10 training trials on task S1. F) Retrograde effects in learning as quantified by the difference in error rates between the end of training task S1 and testing on S1 after training on all tasks. Note that facilitation effects correspond to positive values while interference effects correspond to negative values in both E) and F).

The performance results of training the SORN on different combinations of tasks similarities and training schedules are shown in Fig 13 separately for anterograde (E) and retrograde effects (F). Additionally, plots (panels A-D) of the error rates of the SORN during training are provided to obtain some intuitions about the observed effects for four combinations of training schedules and task similarities, specifically: randomly interleaved training with non-overlapping sequences (A), blocked training with non-overlapping sequences (B), randomly interleaved training with sequences with task similarity of 0.875 (C), blocked training with sequences with task similarity of 0.875 (D). First of all, plot E and F demonstrate that across all combinations one can find regions in the space of task similarity and training schedule that show all reported facilitation and interference effects, both anterograde as well as retrograde. While retrograde effects are primarily dominated by the training schedule, anterograde effects show a pattern of interaction between training schedule and task similarity. Closer examination of the map of anterograde effects (E) shows, that interference effects are associated with more blocked training schedules at low task similarities. With more randomized training schedules and with higher task similarities the interference effects diminish and then turn into facilitation effects. The retrograde effects depicted in panel (F) show strong retrograde interference for blocked training across a wide range of tasks similarities. These interference effects disappear at intermediate training schedules, i.e. when training with more than 30 blocks with 20 repetitions of a each sequence in a single block. Further reducing the number of repetitions in a single block finally leads to retrograde facilitation effects. In summary, these experiments provide evidence that task similarity and training schedule interact to give rise to proactive and retroactive interference and facilitation effects.

## Discussion

In this work we have shown how different phenomena in human sequence learning can all be understood based on generic learning principles in a recurrent neural network model. Specifically, we have considered a sparsely connected recurrent network whose activity and connectivity is shaped by three plasticity mechanisms: spike-timing dependent plasticity (STDP), an intrinsic plasticity regulating neuronal excitability, and a synaptic normalization controlling the amount of afferent input to each neuron. The network receives stimulus-specific input and is connected to a layer of “motor” neurons mediating the movement sequences through a winner-take-all mechanism. We have used this network to model a series of experiments on movement sequence learning using a single set of parameters in all simulations. The network learns to carry out the correct movement sequences over trials and reproduces differences in behavior between training schedules such as blocked vs. randomly interleaved training. The network also shows close similarity to human performance in tasks with similar training sequences but different training times.

Like various pervious models [5–9], our model of sequence learning is formulated as a spiking network learning through STDP and we have used it to model behavioral data from human subjects. We view this approach as complementary to recent modeling efforts using firing rate networks to reproduce neural firing patterns in motor cortex, e.g., [41, 42]. Whether such firing patterns can also be learned with spiking networks through (reward-modulated) STDP is an interesting topic for future research, as is the question wether such rate models, often trained with very different learning mechanisms, can reproduce the kinds of behavioral data on interference and facilitation effects that have been the focus of the present study.

The current work presents a detailed analysis of the underlying changes in the neuronal representations of the motor sequences across learning. Mutual information, PCA of network activity, and measures of neuronal selectivity reveal how neural activity changes with training and how these changes crucially depend on the three plasticity mechanisms in the SORN. Finally, we have provided testable predictions for future experiments jointly varying task similarity and training schedule. Overall, we have shown how task similarity and training schedule can interact to produce a rich set of interference and facilitation effects thereby unifying procedural memory consolidation and structure learning in a recurrent network model with multiple plasticity mechanisms.

The SORN model we have used in this study is admittedly a gross simplification of learning processes in real cortical networks. It uses binary threshold units operating in discrete time steps and highly abstracted forms of plasticity. It is intriguing, however, that networks from the SORN family have already managed to account for both various structural features of cortical networks [22, 43], as well as a large range of physiological findings on neural variability and the relationship between spontaneous and evoked activity [44]. This suggests that despite their simplicity they capture some essential aspects of cortical information processing and learning. Therefore, it is maybe not that surprising that they also manage to account for a range of psychophysical findings on human sequence learning as we have demonstrated here.

Studying the restructuring of neural circuits and their changes in representation during sequence learning in human subjects is currently not feasible. However, extended recordings from the same neural circuit during acquisition of a complex behavior are now possible in animal experiments. Impressively, [45] have even optogenetically reversed synaptic changes occurring during learning of a motor task thereby erasing a recently acquired engram. This makes studying the neural mechanisms underlying sequence learning behaviors both experimentally and theoretically a promising direction for future research.

## Supporting information

S1 Fig (A) Simulation of experimental group of the Panzer et al. [24] study. Sum of pairwise distance of network states under different input conditions using Euclidean norm. The network connection probability between excitatory neurons was set to *p*_*connect*_ = 0.05.
(EPS)

S2 Fig (A) Simulation of experimental group of the Panzer et al. [24] study. Sum of pairwise distance of network states under different input conditions using Euclidean norm. The network connection probability between excitatory neurons was set to *p*_*connect*_ = 0.1. This is the value used in the simulations of the main text.
(EPS)

S3 Fig (A) Simulation of experimental group of the Panzer et al. [24] study. Sum of pairwise distance of network states under different input conditions using Euclidean norm. The network connection probability between excitatory neurons was set to *p*_*connect*_ = 0.15.
(EPS)

S4 Fig (A) Simulation of experimental group of the Panzer et al. [24] study. Sum of pairwise distance of network states under different input conditions using Euclidean norm. The network connection probability between excitatory neurons was set to *p*_*connect*_ = 0.2.
(EPS)

S5 Fig (A) Simulation of experimental group of the Panzer et al. [24] study. Sum of pairwise distance of network states under different input conditions using Euclidean norm. The ratio of excitatory to inhibitory neurons was set to *N*_*I*_/*N*_*E*_ = 0.1.
(EPS)

S6 Fig (A) Simulation of experimental group of the Panzer et al. [24] study. Sum of pairwise distance of network states under different input conditions using Euclidean norm. The ratio of excitatory to inhibitory neurons was set to *N*_*I*_/*N*_*E*_ = 0.2. This is the value used in the simulations of the main text.
(EPS)

S7 Fig (A) Simulation of experimental group of the Panzer et al. [24] study. Sum of pairwise distance of network states under different input conditions using Euclidean norm. The ratio of excitatory to inhibitory neurons was set to *N*_*I*_/*N*_*E*_ = 0.4.
(EPS)
